# Supplementary material for: Frequency of Diarrheagenic Virulence Genes and Characteristics in Escherichia coli Isolates from Pigs with Diarrhea in China
Source: Microorganisms. 2019 Sep 2;7(9):308. doi: 10.3390/microorganisms7090308 (PMC6780709; doi:10.3390/microorganisms7090308)
Supplement: Supplementary file 1 [file microorganisms-07-00308-s001.zip › microorganisms-574093- revised sup-1/Supplementary Figure 1R.docx]

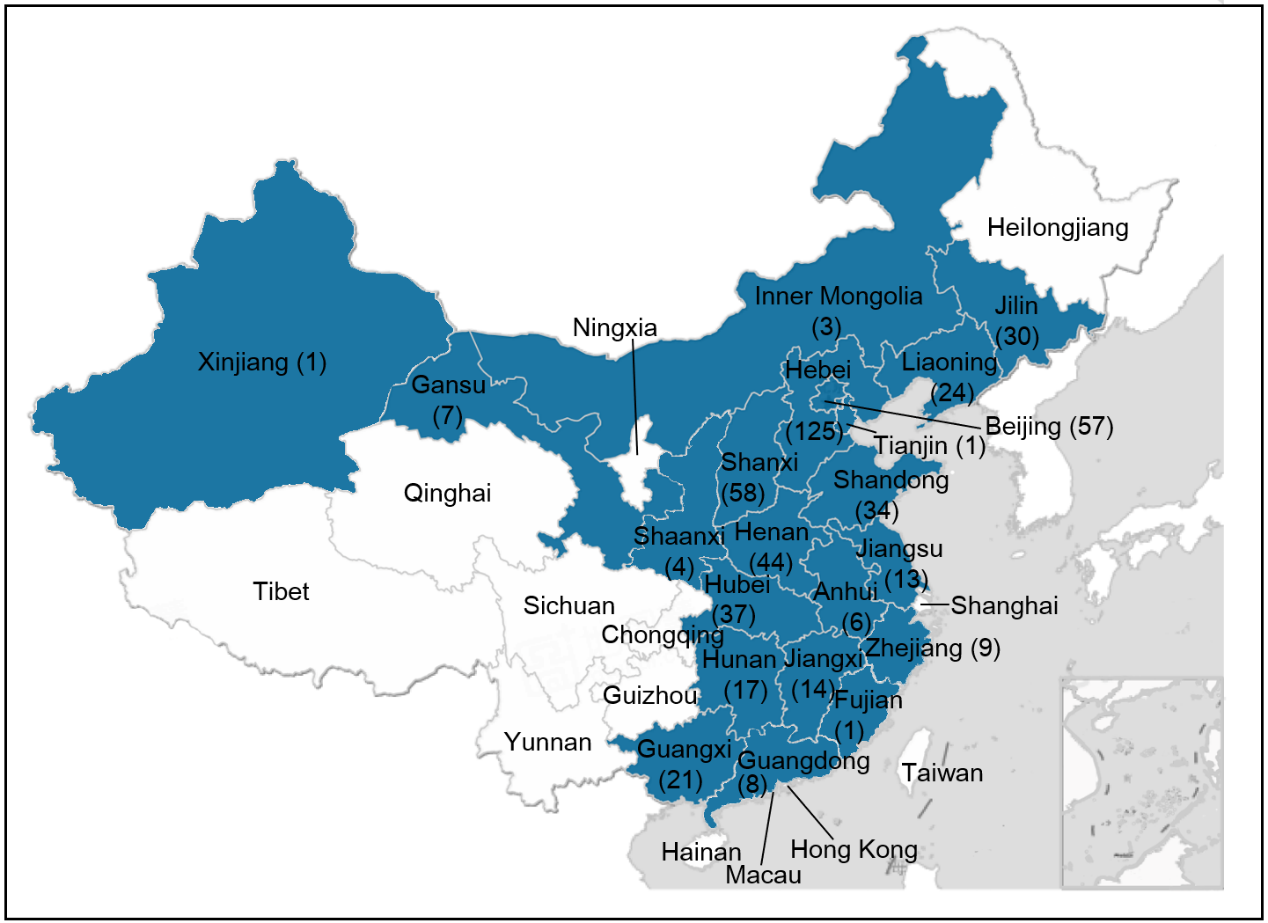


**Supplementary Figure S1: Map of China**. Blue area indicates the distribution of intestinal pathogenic *E. coli* isolates from pigs with diarrhea in Chinese large-scale swine farms from 2014 to 2016. These swine farms were randomized when there was diarrhea outbreak. Numbers in these provinces indicate the number of *E. coli* strains we used in the current study.
