# Supplementary material for: Frequency of Diarrheagenic Virulence Genes and Characteristics in Escherichia coli Isolates from Pigs with Diarrhea in China
Source: Microorganisms. 2019 Sep 2;7(9):308. doi: 10.3390/microorganisms7090308 (PMC6780709; doi:10.3390/microorganisms7090308)
Supplement: Supplementary file 1 [file microorganisms-07-00308-s001.zip › microorganisms-574093- revised sup-1/Supplementary Figure 2.pdf]

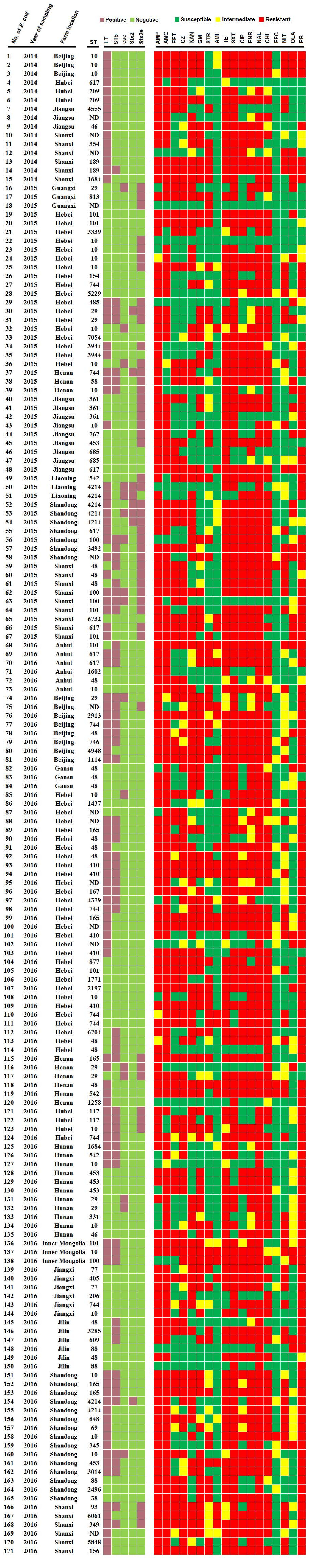

Positive
Negative
Susceptible
Intermediate
Resistant
No. of E. coli
Year of sampling
Farm location
ST
LT
STb
eae
Stx2
Stx2e
AMP
AMC
EFT
CZ
KAN
GM
STR
AMI
TE
SXT
CIP
ENR
NAL
CHL
FFC
NIT
OLA
PB
1 2014 Beijing 10
2 2014 Beijing 10
3 2014 Beijing 10
4 2014 Hubei 617
5 2014 Hubei 209
6 2014 Hubei 209
7 2014 Jiangsu 4555
8 2014 Jiangsu ND
9 2014 Jiangsu 46
10 2014 Shanxi ND
11 2014 Shanxi 354
12 2014 Shanxi ND
13 2014 Shanxi 189
14 2014 Shanxi 189
15 2014 Shanxi 1684
16 2015 Guangxi 29
17 2015 Guangxi 813
18 2015 Guangxi ND
19 2015 Hebei 101
20 2015 Hebei 101
21 2015 Hebei 3339
22 2015 Hebei 10
23 2015 Hebei 10
24 2015 Hebei 10
25 2015 Hebei 10
26 2015 Hebei 154
27 2015 Hebei 744
28 2015 Hebei 5229
29 2015 Hebei 485
30 2015 Hebei 29
31 2015 Hebei 29
32 2015 Hebei 10
33 2015 Hebei 7054
34 2015 Hebei 3944
35 2015 Hebei 3944
36 2015 Hebei 10
37 2015 Henan 744
38 2015 Henan 58
39 2015 Henan 10
40 2015 Jiangsu 361
41 2015 Jiangsu 361
42 2015 Jiangsu 361
43 2015 Jiangsu 10
44 2015 Jiangsu 767
45 2015 Jiangsu 453
46 2015 Jiangsu 685
47 2015 Jiangsu 685
48 2015 Jiangsu 617
49 2015 Liaoning 542
50 2015 Liaoning 4214
51 2015 Liaoning 4214
52 2015 Shandong 4214
53 2015 Shandong 4214
54 2015 Shandong 4214
55 2015 Shandong 617
56 2015 Shandong 100
57 2015 Shandong 3492
58 2015 Shandong ND
59 2015 Shanxi 48
60 2015 Shanxi 48
61 2015 Shanxi 48
62 2015 Shanxi 100
63 2015 Shanxi 100
64 2015 Shanxi 101
65 2015 Shanxi 6732
66 2015 Shanxi 617
67 2015 Shanxi 101
68 2016 Anhui 101
69 2016 Anhui 617
70 2016 Anhui 617
71 2016 Anhui 1602
72 2016 Anhui 48
73 2016 Anhui 10
74 2016 Beijing 29
75 2016 Beijing ND
76 2016 Beijing 2913
77 2016 Beijing 744
78 2016 Beijing 48
79 2016 Beijing 746
80 2016 Beijing 4948
81 2016 Beijing 1114
82 2016 Gansu 48
83 2016 Gansu 48
84 2016 Gansu 48
85 2016 Hebei 10
86 2016 Hebei 1437
87 2016 Hebei ND
88 2016 Hebei ND
89 2016 Hebei 165
90 2016 Hebei 48
91 2016 Hebei 48
92 2016 Hebei 48
93 2016 Hebei 410
94 2016 Hebei 410
95 2016 Hebei ND
96 2016 Hebei 167
97 2016 Hebei 4379
98 2016 Hebei 744
99 2016 Hebei 165
100 2016 Hebei ND
101 2016 Hebei 410
102 2016 Hebei ND
103 2016 Hebei 410
104 2016 Hebei 877
105 2016 Hebei 101
106 2016 Hebei 1771
107 2016 Hebei 2197
108 2016 Hebei 10
109 2016 Hebei 410
110 2016 Hebei 744
111 2016 Hebei 744
112 2016 Hebei 6704
113 2016 Hebei 48
114 2016 Hebei 48
115 2016 Henan 165
116 2016 Henan 29
117 2016 Henan 29
118 2016 Henan 48
119 2016 Henan 542
120 2016 Henan 1258
121 2016 Hubei 117
122 2016 Hubei 117
123 2016 Hubei 10
124 2016 Hubei 744
125 2016 Hunan 1684
126 2016 Hunan 542
127 2016 Hunan 10
128 2016 Hunan 453
129 2016 Hunan 453
130 2016 Hunan 453
131 2016 Hunan 29
132 2016 Hunan 29
133 2016 Hunan 331
134 2016 Hunan 10
135 2016 Hunan 46
136 2016 Inner Mongolia 101
137 2016 Inner Mongolia 10
138 2016 Inner Mongolia 100
139 2016 Jiangxi 77
140 2016 Jiangxi 405
141 2016 Jiangxi 77
142 2016 Jiangxi 206
143 2016 Jiangxi 744
144 2016 Jiangxi 10
145 2016 Jilin 48
146 2016 Jilin 3285
147 2016 Jilin 609
148 2016 Jilin 88
149 2016 Jilin 48
150 2016 Jilin 88
151 2016 Shandong 10
152 2016 Shandong 165
153 2016 Shandong 165
154 2016 Shandong 4214
155 2016 Shandong 4214
156 2016 Shandong 648
157 2016 Shandong 69
158 2016 Shandong 10
159 2016 Shandong 345
160 2016 Shandong 10
161 2016 Shandong 453
162 2016 Shandong 3014
163 2016 Shandong 88
164 2016 Shandong 2496
165 2016 Shandong 38
166 2016 Shanxi 93
167 2016 Shanxi 6061
168 2016 Shanxi 349
169 2016 Shanxi ND
170 2016 Shanxi 5848
171 2016 Shanxi 156

Supplementary Figure S2: Data regarding STs, virulence genes, and antimicrobial resistance profile of *E. coli* isolates obtained from pigs with diarrhea between 2014 and 2016. AMP, ampicillin; AMC, amoxicillin-clavulanate; EFT, ceftiofur; CZ, cefazolin; KAN, kanamycin; GM, gentamicin; STR, streptomycin; AMI, amikacin; TE, tetracycline; SXT, trimethoprim-sulfamethoxazole; CIP, ciprofloxacin; ENR, enrofloxacin; NAL, nalidixic acid; CHL, chloramphenicol; FFC, florfenicol; NIT, nitrofurantoin; OLA, olaquindox; PB, polymyxin B. ND, not determined.
